# Correlation of disease severity with body weight and high fat diet in the FATZO/Pco mouse

**DOI:** 10.1371/journal.pone.0179808

**Published:** 2017-06-22

**Authors:** Brian A. Droz, Bria L. Sneed, Charles V. Jackson, Karen M. Zimmerman, M. Dodson Michael, Paul J. Emmerson, Tamer Coskun, Richard G. Peterson

**Affiliations:** 1 Eli Lilly and Company, Indianapolis, Indiana, United States of America; 2 Ball State University, Muncie, Indiana, United States of America; 3 Crown Bioscience - Indiana, Indianapolis, Indiana, United States of America; East Tennessee State University, UNITED STATES

## Abstract

Obesity in many current pre-clinical animal models of obesity and diabetes is mediated by monogenic mutations; these are rarely associated with the development of human obesity. A new mouse model, the FATZO mouse, has been developed to provide polygenic obesity and a metabolic pattern of hyperglycemia and hyperinsulinemia, that support the presence of insulin resistance similar to metabolic disease in patients with insulin resistance/type 2 diabetes. The FATZO mouse resulted from a cross of C57BL/6J and AKR/J mice followed by selective inbreeding for obesity, increased insulin and hyperglycemia. Since many clinical studies have established a close link between higher body weight and the development of type 2 diabetes, we investigated whether time to progression to type 2 diabetes or disease severity in FATZO mice was dependent on weight gain in young animals. Our results indicate that lighter animals developed metabolic disturbances much slower and to a lesser magnitude than their heavier counterparts. Consumption of a diet containing high fat, accelerated weight gain in parallel with disease progression. A naturally occurring and significant variation in the body weight of FATZO offspring enables these mice to be identified as low, mid and high body weight groups at a young age. These weight groups remain into adulthood and correspond to slow, medium and accelerated development of type 2 diabetes. Thus, body weight inclusion criteria can optimize the FATZO model for studies of prevention, stabilization or treatment of type 2 diabetes.

## Introduction

The incidence of obesity is increasing at an alarming rate, with the prevalence in adolescence especially concerning [1,2]. Obesity is characterized as a chronic inflammatory state [3] and as such, a relationship between obesity and insulin resistance [4,5], Alzheimer’s disease [6], atherosclerosis [7] and poor pregnancy outcomes [8–10] has been identified. Obesity is an independent risk factor for the development of type 2 diabetes [11–13] and cardiovascular disease [14–19]. Indeed, type 2 diabetes and obesity have been termed the twin pandemics [20]. Many clinical studies have established a close link between higher body weight and type 2 diabetes [21]. In patients identified as pre-diabetic, weight loss is considered first line intervention to reduce the risk of progression to type 2 [22,23]. The necessity for anti-diabetic therapy was negated in patients with type 2 diabetes with sustained weight loss, and reduction in body weight has been shown to improve glycemic control [24]. In addition, body weight is a strong predictor for the development of complications of diabetes [25–27], and weight loss following bariatric surgery or very low calorie diets have proven beneficial in glucose control in type 2 diabetes [28].

Obesity in many currently used pre-clinical animal models, is mediated by a monogenic disruption in leptin signaling [29–34] or is initiated by high fat feeding (DIO models) [32,35,36]. A monogenic cause for obesity is rare in humans [37]. Thus, while obesity induces diabetes in these models, the mechanism eliciting obesity is quite different. Consumption of a diet high in fat (HFD) definitely contributes to obesity and type 2 diabetes in patients [38–40], as it does in DIO animals. However, DIO animals do not typically develop severe hyperglycemia necessary for the study of severe disease or the complications that develop as a result of severe disease [30,32–34,41].

The FATZO mouse was developed by crossing two commonly used DIO models the C57BL/6 and the AKR/J [32,35,36,42–46] followed by selective inbreeding to genetic homogeneity (30+ generations). The selection of higher body weight animals for breeding was preferred to promote obesity; however, excessive body weight resulted in lower pregnancy rates, smaller liters and reduced survival of offspring. The result of this selection process was effective, but not optimal and has led to a significant variation in body weight of FATZO offspring when fed normal chow diet. This variation is apparent at weaning and carries through to adulthood. Independent of initial body weight, abnormal glucose disposal is apparent compared to control mice [47]. Variations in body weight for DIO mice have also been reported, due to outliers, in response to the high fat diet. It is estimated that 10% of mice do not gain sufficient weight when eating the high fat diet (www.jax.org), necessitating a body weight inclusion criteria to ensure an obese phenotype for DIO studies.

The aim of this study was to compare the effects of chow and a high fat diet on the development of obesity and diabetes in the FATZO mouse.

## Materials and methods

### Ethics statement

The PCO now CBIN (Crown Bioscience—Indiana) and Eli Lilly and Company’s Institutional Animal Care and Use Committees approved animal experiments.

### FATZO production

FATZO mice in the breeding colony were maintained on Purina 5008 rodent diet and reverse osmosis water. Mice were bred between 6 and 10 weeks of age (optimally 7–8 weeks old). Animals were housed in a light (12hr light/ 12 hr dark) and temperature (25°C) controlled environment.

### The effect of high fat diet

Male FATZO/Pco mice (n = 48) were weighed (24–43 g) at 6 weeks of age and transferred from the CBIN colony (Crown Bioscience—Indiana, Indianapolis, Indiana, USA) to Lilly Research Laboratories (Indianapolis, IN, USA) at 6–8 weeks of age. After acclimation, FATZO mice averaging 10 weeks of age were assigned to groups based on their 6-week weights as follows: Low BW (low weight, 23–26.9g), Mid BW (mid weight, 27–29.9g) and High BW (high weight, ≥30g). At the initiation of the study, the average weights of the groups of 10 week old mice were: Low BW (29.4 ± 0.7 g), Mid BW (35.7 ± 0.7 g) and High BW (38.9 ± 0.7 g). Mice in each weight group were randomized into 2 subgroups (n = 8/subgroup) that were fed either Purina 5008, 16% fat chow (Chow) or D12492, 60% fat diet from Research Diets (HFD, New Brunswick, NJ, USA). Body weight was recorded weekly; whole blood glucose (AccuChek Aviva meters) levels were recorded weekly from 10 to 18 weeks of age and again at 21 weeks of age. Blood was collected from mice at 10, 12, 14, 18 and 21 weeks of age and plasma was prepared for insulin analysis. Blood samples for glucose and insulin were obtained by tail snip in the fed state. An oral glucose tolerance test (OGTT) was performed following a 17 hour fast in 18 week old mice to assess glucose disposal; glucose and insulin levels were assayed from samples taken at 0, 15, 30, 60 and 120 min post-glucose load (3 g/kg, PO). Plasma from blood samples collected throughout the study and during the OGTTs was analyzed for insulin using the mouse/rat insulin assay kit (K152BZC, Meso Scale Discovery, Rockville, MD, USA).

Animals were euthanized with CO_2_ at 21 weeks of age. Each pancreas was dissected, weighed, snap frozen in liquid N_2_ and placed in EtOH-HCl (5 ml) extraction buffer (23.5 parts water, 75 parts ethanol, 1.5 parts concentrated HCl) and kept at 4°C. After thorough mincing with a polytron homogenizer, the pancreas was extracted in the buffer by overnight shaking at 4°C. The tissue was separated from the extract by centrifugation and diluted for insulin analysis with Earle's Balanced Salt Solution (EBSS) with 0.1% BSA. These extracts were also analyzed using the mouse/rat insulin assay kit (K152BZC, Meso Scale Discovery, Rockville, MD, USA).

### Leptin levels and the effect of leptin on food intake

Two age groups of male FATZO mice were selected for leptin levels and the effect of leptin on food intake. Additional age matched groups of C57BL/6 were bled for leptin levels. Animals were acclimated to reverse light cycle for 7 days before being put on protocol. Thirty to sixty minutes before lights were turned out, blood was collected for leptin levels from 5 (N = 7) and 11 (N = 8) week old male FATZO mice and from male C57BL/6 at approximately the same ages (N = 6). Serum was prepared from tail blood and leptin levels were analyzed (Meso Scale Discovery, K152BYC, Rockville, MD, USA). After blood collection, animals were given saline or leptin injections (10 mg/kg) and food intake data was recorded for the first 4 hours of darkness.

### Statistics

Except where mentioned, all data are presented as Mean ± SEM. Statistical analysis was done using Prism for Windows (version 6.07 GraphPad, San Diego, CA, USA). When comparing groups, one-way ANOVA followed by Sidak’s multiple comparisons test were done; two-way ANOVA followed by Sidak’s multiple comparison tests were performed when groups were compared over time. Linear regression was performed on paired (6-week body weight versus 14-week glucose and insulin) followed by a correlation analysis.

## Results

### FATZO production

Development and breeding of the FATZO mouse model required some special conditions. Based on the rapid weight gain of these animals, the breeding ages are more limited than with usual mouse colonies. Breeding was most successful when done between 7–8 weeks of age. Successful litters from lower body weight animals could be produced over a longer period of time; however, this practice resulted in lower body weight offspring and increased the potential for drift towards a less obese model. Older, heavier animals could be bred, but this resulted in fewer pregnancies and lower production. The breeding and housing at higher temperatures was also an important consideration since higher temperatures, closer to thermo-neutral, enhanced weight gain and disease expression. An analysis of 53 litters of FATZO mice indicated a negative correlation of litter size with male pup body weights (r = -0.6986, p<0.0001). Since larger litters contained lower body weight animals, restricting litter size resulted in heavier offspring. Despite normalization of litter size, variation in animal weights was still observed.

### Animal weights

**Fig 1:**
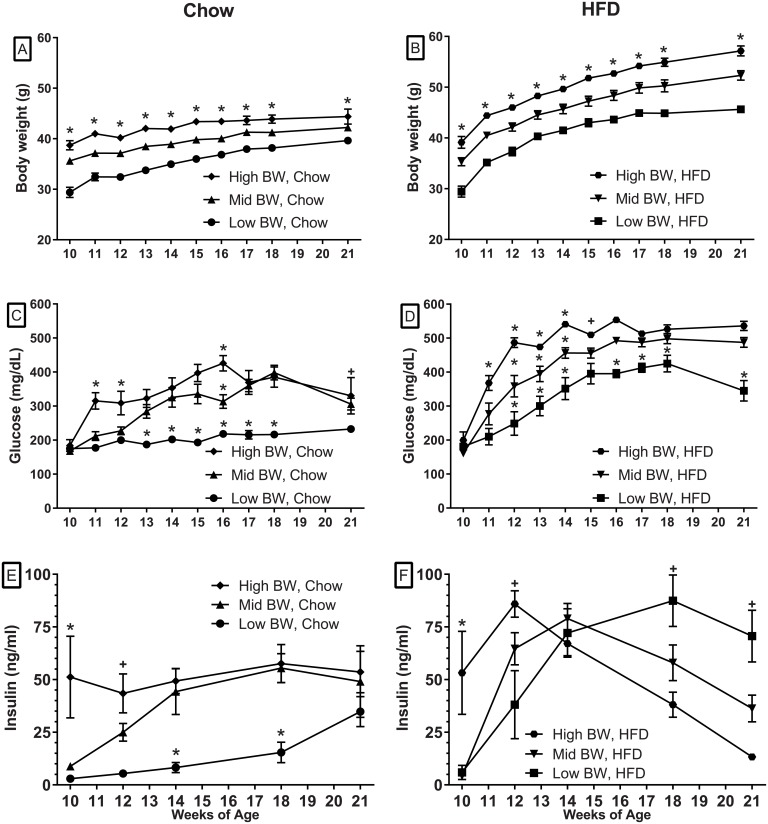
The effects of initial weight and diet on body weight, glucose and insulin levels. The top figures illustrate Body weight gains of animals on the Chow diet (A) and HFD (B). The middle panels (C, D) demonstrate the effects of the two diets on glucose levels. Insulin levels in Chow (E) and HFD (F) groups are illustrated in bottom panels. The * (A, B) indicates that all weight groups are statistically different from each other at all of the time-points with each diet. Statistical differences from the other groups (C-F) are identified by * while the ^+^ sign identifies differences between the highest and lowest values (two-way ANOVA followed by Sidak’s multiple comparison test, * or ^+^ *p* < .05). Additional differences between diets in each weight category are summarized in the text.

Animals were grouped based on their 6-week (initial) weights to determine the influence of early weight on subsequent weight gain, insulin levels, glucose tolerance and hyperglycemia. The averages of the weight groups within each diet remained significantly different from each other at all time-points with both Chow and HFD diets (Fig 1A and 1B). The mice fed the HFD (Fig 1B) all gained weight more rapidly than the weight-matched, Chow-fed animals (Fig 1A). Two-way RM ANOVA demonstated significant differences in weight between the Chow and HFD with each weight category at every time point after diet initiation (*p* < .0005). Table 1 demonstrates that all weight groups of mice on HFD ate less mass of the diet than the animals on Chow. However, the High BW, HFD group had higher caloric intake compared to the High BW Chow fed group.

**Table 1:** Food consumption.

	Cumulative Food Consumption (g)	Cumulative Caloric Intake (kcal)
**Low BW, Chow**	230.6 ±4.2, n = 8	807.1 ±14.8, n = 8
**Low BW, HFD**	159.4 ±3.3, n = 8*	835.2 ±17.5, n = 8
**Mid BW, Chow**	241.9 ±4.8, n = 8	846.5 ±16.7, n = 8
**Mid BW, HFD**	172.4 ±5.9, n = 8*	903.4 ±30.8, n = 8
**High BW, Chow**	268.6 ±8.1, n = 7	940.0 ±28.3, n = 7
**High BW, HFD**	204.8 ±3.2, n = 8*	1073.3 ±16.9, n = 8*

* indicates statistical differences between Chow and HFD in the weight group.

### Glucose levels

Despite similar glucose levels between the weight groups at 10 weeks of age (Fig 1C and 1D), glucose differences became evident over time in both the Chow and HFD groups. The initial rise in glucose levels for both the Chow (Fig 1C) and HFD (Fig 1D) groups correlated with initial body weight. The Chow fed, High BW and Mid BW groups had comparable increased glucose levels from 13 to 21 weeks of age while Low BW Chow group remained at close to baseline levels for the duration of the experiment (Fig 1C). Similarly, the 2 heaviest groups of HFD animals attained the highest glucose levels over time, while the low BW group had significantly lower glucose levels over the course of the experiment (Fig 1D). As with weight, two-way RM ANOVA identified significant differences in glucose curves between the Chow and HFD with each weight category (*p* < .005). Sidak's multiple comparisons test also demonstrated significant differences between the diets at all ages (*p* < .01) except at 10–12 weeks of age in the Low BW groups and at 10, 11, 15 and 18 weeks in the Mid BW and 10 and 11 weeks in the High BW Groups.

### Insulin levels

The results demonstrate a positive relationship between body weight and plasma insulin levels in the 10–12 week data. Initial 10-week insulin levels of the High BW groups were significantly higher than the two lower body weight groups (Fig 1E and 1F). In the Chow fed/High BW group, the average insulin levels did not change over time (Fig 1E). However, in both the Mid BW and Low BW groups, insulin levels increased over time with the Mid BW group becoming similar to the High BW group at 14 weeks of age and the Low BW group at 21 weeks (Fig 1E). In contrast, the insulin levels in the HFD groups increased dramatically over the first 4 weeks of HDF with the average insulin levels becoming similar at 14 weeks of age. Subsequently, the insulin levels decreased in the two higher weight groups (Fig 1F).

### Glucose tolerance

**Fig 2:**
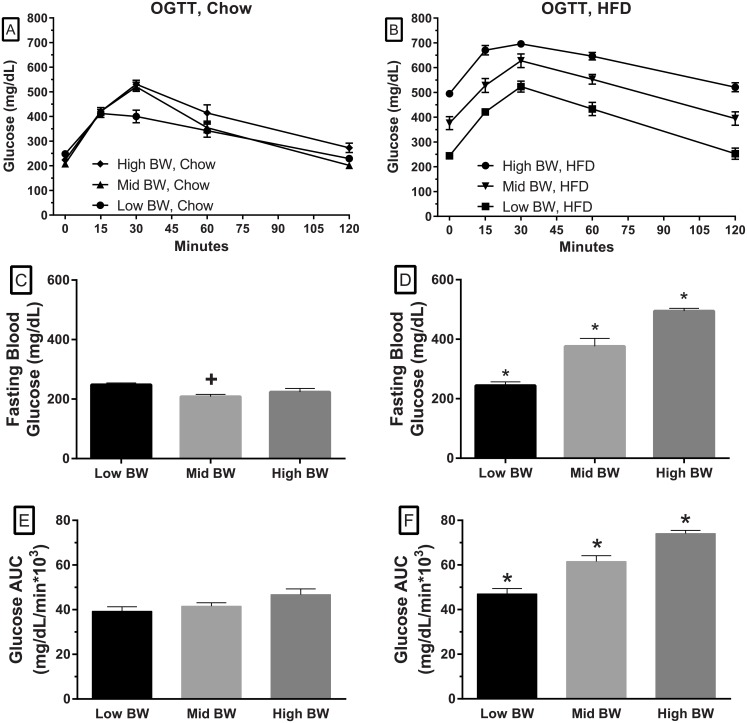
The effects of initial weight and diet on fasting glucose and glucose disposal in an OGTT. The top figures show glucose responses in the OGTT for mice fed the Chow diet (A) and the HFD. Baseline glucose levels, after a 17 hour fast in the Low, Mid and High BW groups of mice fed the Chow diet (C) while glucose levels are significantly higher in the Mid BW and High BW animals on the HFD (D). The figures in the two lower panels illustrate the glucose AUC from the OGTT for animals in each of the body weight groups when fed Chow diet (E) and HFD (F). (one-way ANOVA followed by Sidak’s multiple comparison tests. ^+^ denotes a statistical difference compared to the Low BW and * denotes statistical from all other group, *p* < .05).

**Fig 3:**
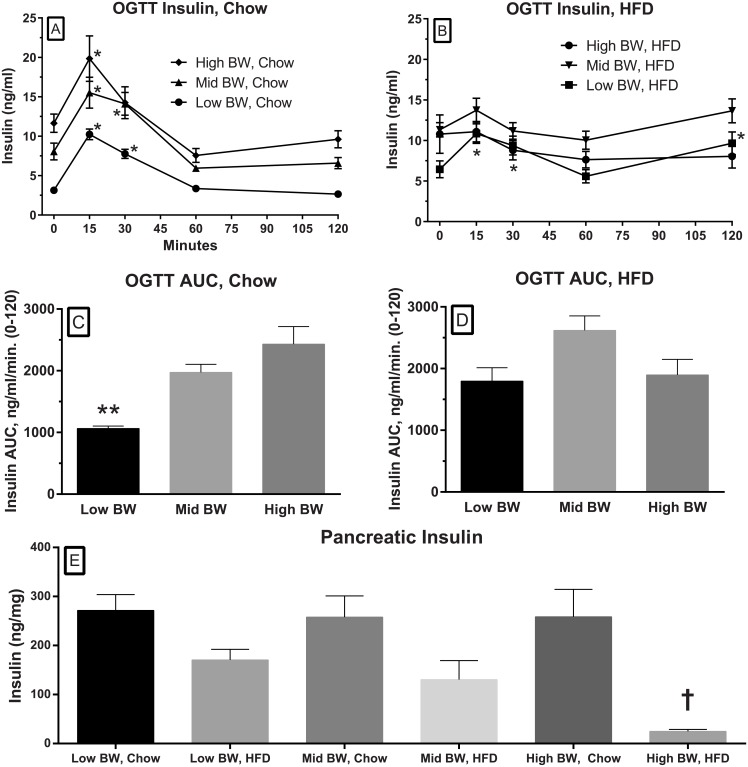
The effects of initial weight and diet on insulin levels during the OGTT. This graph illustrates the insulin response to a glucose load at 18 weeks in the Low BW, Mid BW and High BW groups when fed the Chow diet (A) or the HFD (B). The * (A, B) indicates significant increases from baseline for that group (two-way ANOVA followed by Sidak’s multiple comparison tests). The insulin AUCs (C, D) for the above OGTTs (A, B) are also illustrated; the ** indicates a significant difference between the Low BW group and the other two groups (one-way ANOVA followed by Sidak’s multiple comparison tests). The lowest panel (E) illustrates the insulin content of the pancreas from the different weight groups on the two diets at 21 weeks. The effect of diets was tested in respective weight pairs (High BW, Mid BW and High BW); † denotes there was a statistically significance difference between the two diets in the High BW groups (one-way ANOVA followed by Sidak’s multiple comparison tests). *, **, † denotes statistical significance at the level *p* < .05).

An OGTT was performed when the animals were about 18 weeks of age (Fig 2A and 2B). Fig 2C and 2D show the fasted glucose levels after an overnight fast. These figures illustrate that fasted glucose levels in the Chow-fed groups are quite similar at baseline (Fig 2C), while they remain significantly elevated in the two heavier HFD groups (Fig 2D). The Chow fed glucose levels in the OGTT showed a significant excursion that was greater than what one would typically see in a control mouse (~30*10^3^ in comparable studies, PCO unpublished) but with the overnight fast, the glucose levels fall close to fasting levels in 120 minutes; the AUC data demonstrated similar values for the three groups (Fig 2E). The Mid and High BW, HFD groups demonstrate elevated fasting glucose levels (Fig 2D) and delayed glucose disposal (Fig 2B and 2F). Serum insulin levels were determined for the time-points in the OGTT. The insulin response in the Chow-fed groups showed a transient increase after the glucose load (Fig 3A) while the animals fed the HFD had a blunted response (Fig 3B). The fasting insulin levels in the Chow-fed groups were increased relative to body weight. The insulin levels in response to the glucose load were significantly increased from baseline at 30 and 60 minutes (Fig 3A). The AUC for insulin during the OGTT in the Chow fed groups (Fig 3C) also demonstrated that there was a relationship between weight and the AUC with the lightest groups having a significantly lower insulin AUC. In the HFD groups, there was no increase from baseline insulin levels in the High and Mid BW groups, but there were significant increases from baseline in the Low BW group (Fig 3B).

### Pancreatic insulin

At the end of the experiment pancreata were removed and insulin content was determined. In accordance with plasma insulin data, pancreatic insulin was also significantly reduced in the HFD, High BW group when compared to the Chow, High BW group (Fig 3E).

### Body weight, glucose and insulin correlation

**Fig 4:**
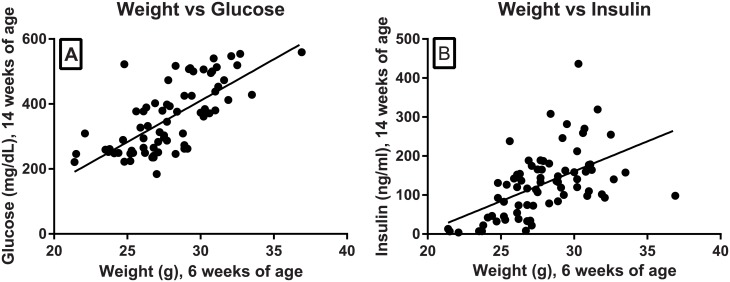
Weight correlated with glucose and insulin. Glucose (A) and insulin (B) levels at 14 weeks were plotted and analyzed according to the animal weights at 6 weeks of age (weight vs glucose, r = 0.7033, *p* < .0001; weight vs insulin, r = 0.5317, *p* < .0001).

Retrospective analysis of a cohort of 73 FATZO male mice demonstrated that glucose and insulin in 14 week old mice were positively correlated with body weights of 6 week old mice (Fig 4).

### Leptin levels and the effect of leptin on food intake

**Fig 5:**
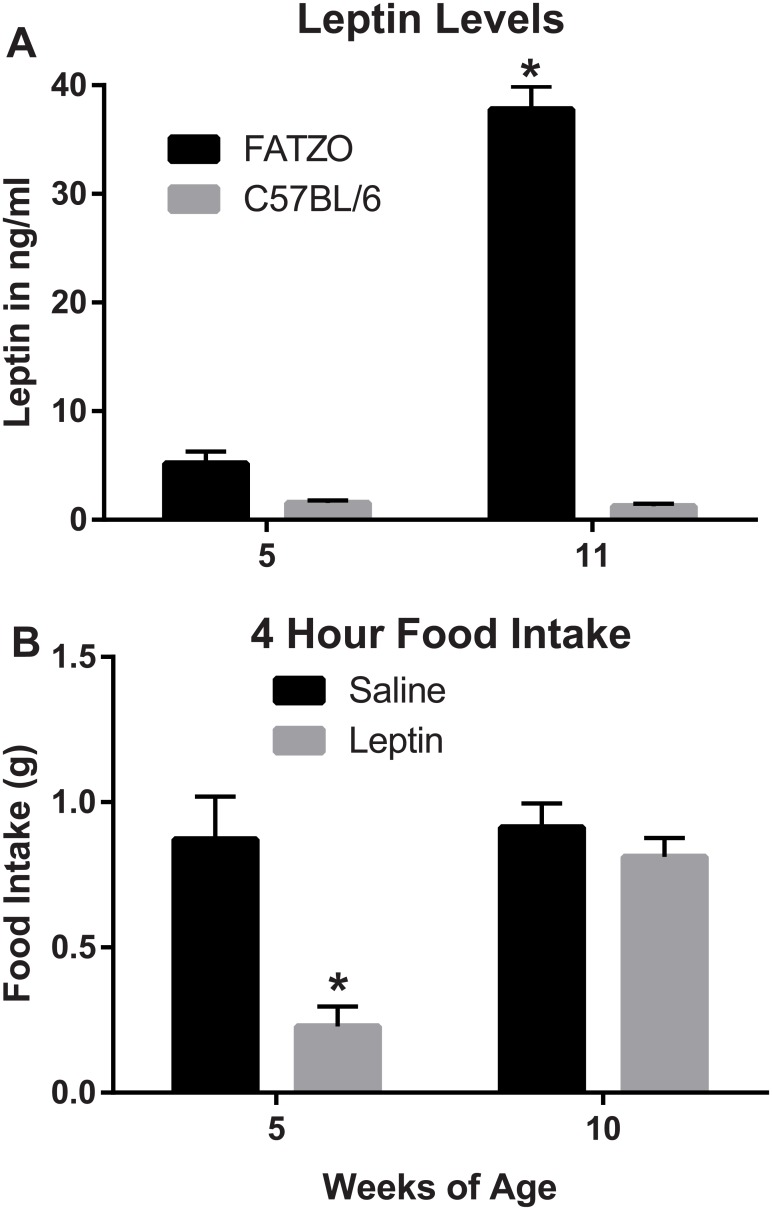
Leptin levels and the effect of leptin on food intake. Leptin levels (A) and the effect of leptin on food intake (B) at two ages are illustrated in this figure (one-way ANOVA followed by Sidak’s multiple comparison tests). Statistical differences from the other groups are denoted at *p* < .001(**) and *p* < .0001(***).

At 5 weeks of age leptin levels are not significantly different between FATZO and C57BL/6 mice but as the FATZO mice become more obese their leptin levels increase rapidly while the C57BL/6 mice remain low and are not significantly different from 5 week-old animals (Fig 5A). Food consumption is significantly reduced with leptin injections in young FATZO animals but as the endogenous leptin levels increase at 10 weeks of age there is no significant reduction in food intake (Fig 5B).

## Discussion

The purpose of developing the FATZO mouse was to create a more translatable model for understanding the physiological and cellular mechanisms that lead from obesity to diabetes. The data presented in this paper demonstrated that the male FATZO mouse has the characteristics that make it a viable model for these purposes. The obesity, high insulin levels and glucose intolerance leading to hyperglycemia, appeared to phenotypically recapitulate the human disease. One of the strengths of the model is the prolonged time during which animals are hyperglycemic without the loss of circulating insulin levels or decreases in pancreatic insulin content. Hyperinsulinemia concurrent with hyperglycemia, as biomarkers of insulin resistance, make this mouse a viable model to study mechanisms leading to increased insulin sensitivity. Although defined beta cell failure was not present, the data presented suggest that the beta cells were not able to respond normally to glucose stimulation particularly when the heaviest mice were given a high fat diet. This study and model development focused on male mice since they we recognized as having consistently elevated glucose when they were over 10 weeks of age. The females are also heavier than control mice and likely have some of the components of metabolic syndrome; however, the initial glucose data in the females lead us to focus on the males.

The FATZO mouse was developed by crossing the AKR/J with the C57BL/6J strains and selectively inbreeding to provide the genetics of insulin resistance and obesity. These two strains are known to develop obesity when fed a high fat diet [42]. As reviewed in the “Introduction” these two strains were chosen in an effort to avert monogenic obesity in the resulting model. Although leptin functioning in the FATZO mouse has not been investigated thoroughly, some observations suggest that the pathway is intact. Firstly, leptin pathway interruption typically results in prominent hyperphagia [37,48–51] which has not been observed in FATZO mice fed normal chow (unpublished data). However, an increase in caloric intake in heavier animals when fed the high fat diet may indicate leptin resistance. Secondly, leptin protein or leptin receptor defects typically exhibit a recessive Mendelian inheritance pattern such as that seen in the ob/ob and db/db models; this pattern has not been observed in FATZO breeding. Additionally, data presented in this manuscript show relatively normal leptin levels in male FATZO mice when they are young but the levels go up significantly as the animals age. The data presented also demonstrate that the food intake can be modulated in younger animals, with lower endogenous leptin levels, but this response is significantly blunted as the serum leptin levels go up and leptin resistance presumably increases.

The characteristics of the FATZO model give it several advantages over the commonly used obese models. The most frequently mentioned obese/diabetic mouse models used in basic research and drug screening are those with leptin pathway defects (*db*/*db* and *ob*/*ob*) and the C57BL/6 DIO model. Since single-gene leptin pathway defects are very rare in the human population [52–56], animals with these defects are not representative of the clinical landscape. The *ob*/*ob* model on the C57BL/6J background, lacks active leptin [48,50,57,58]. It has large islets which respond by releasing insulin with glucose elevations. This gene disruption on the C57BL/6J background has been characterized as a “model for the prediabetic state” with beta cell proliferation, hyperphagia, hyperinsulinemia, hyperglycemia, reduced metabolism and depressed thermoregulatory capacity [57]. Since *ob/ob* mice do not exhibit beta cell failure and at older ages actually have reduced glucose levels they have limited usefulness as a model for testing antidiabetic compounds [59]. The *db/db* mutation on the C57BL/Ks has a dysfunctional leptin receptor [37]. This mutation on the Ks background results in obesity and a very early onset of hyperglycemia with beta cell failure. These characteristics result in a very severe model of diabetes and beta cell failure. The rapidity of beta cell failure in the db/db model [60–63] limits its usefulness in studying drugs that modulate beta cell health and the effectiveness of native pancreatic insulin. In both the *ob/ob* and *db/db* models, defects in leptin signaling also interfere with the normal feedback mechanisms to the hypothalamus that are responsible for the control of body weight, feeding and energy expenditure [64,65]. Thus these models are ineffective for testing compounds designed to modulate mechanisms mediated through the CNS. The DIO model, with intact leptin signaling, has been successfully used to demonstrate the effects of excessive caloric intake on obesity. Although the DIO model exhibits obesity, insulin resistance and glucose intolerance, modest glucose levels narrow the treatment window for testing the effects of anti-hyperglycemic compounds [32].

In contrast, the male FATZO mouse gains weight rapidly without identified obesity mutations or special diets. High insulin and hyperglycemia are prominent in this model by 10–14 weeks of age. Metformin, rosiglitazone and semaglutide were effective in treating the insulin resistance and hyperglycemia [47]. Although all of these characteristics are present in FATZO mice fed normal chow diets, the data presented in this investigation demonstrate that the high fat diet (D-12492) used in DIO studies enhanced these characteristics and produced more severe obesity, hyperinsulinemia and hyperglycemia. HFD treatment resulted in significant increases in fasting glucose levels and the area under the curve in the OGTT. These changes in the two heavier groups are likely due to changes in the insulin content of the pancreas or in the function of the beta cells. Further studies need to be done to determine the specific mechanism.

In conclusion, the FATZO mouse exhibited dysfunctional glucose homeostasis in a wide range of severities based on body weight. Within an age group, leaner animals exhibited impaired glucose handling while heavier animals generally displayed more severe glucose intolerance. This phenotypic variability enables the selection of animals in the desired stage of metabolic syndrome/type 2 diabetes. Body weight inclusion criteria can be used to design studies directed at slowing progression (Mid BW) or treatment of (High BW) diabetes. The FATZO mouse is proposed as a novel animal model for the study of obesity/metabolic syndrome and its progression. The glucose stimulated insulin release was blunted in all weight groups fed HFD. This lack of response after a glucose load suggests that the pancreatic beta cells have lost their ability to respond effectively to elevated glucose. Introduction of a high fat diet promotes the development of more severe diabetes characterized by hyperglycemia, decreased insulin release and sensitivity which could ultimately lead to beta cell failure.
